# Promoting health and safety in public transportation: A call to action for sub-Saharan Africa

**DOI:** 10.4102/phcfm.v17i1.4570

**Published:** 2025-04-30

**Authors:** Gugu G. Mchunu, Dudu G. Sokhela, Yugan Pillay, Ivan Niranjan, Celenkosini T. Nxumalo

**Affiliations:** 1 Faculty of Health Sciences, Durban University of Technology, Durban, South Africa; 2 Department of Nursing, Faculty of Health Sciences, Durban University of Technology, Durban, South Africa; 3 Department of Emergency Medical Care and Rescue, Faculty of Health Sciences, Durban University of Technology, Durban, South Africa; 4 Department of Community Health Studies, Faculty of Health Sciences, Durban University of Technology, Durban, South Africa; 5 Research Development and Postgraduate Support, Office of the DVC Research and Innovation, University of the Western Cape, Cape Town, South Africa; 6 Discipline of Nursing School of Nursing and Public Health, University of KwaZulu-Natal, Durban, South Africa

**Keywords:** health and safety, minibus taxi, road traffic incident, road traffic mortality, road traffic morbidity

## Abstract

Road Traffic Incidents (RTIs) are a major public health concern worldwide, particularly in low-income to middle-income regions such as sub-Saharan Africa. Data from sub-Saharan Africa suggests that the public transport industry accounts for the majority of fatal crashes that contribute to the high mortality and morbidity associated with RTIs. In this viewpoint, we advocate for integrated and comprehensive evidence-based health and safety interventions to address the challenge of RTIs in the public transport industry in sub-Saharan Africa. We provide evidence on the magnitude of the problem drawing on the state of mortality and morbidity and reported challenges associated with RTIs in the minibus taxi industry in South Africa as this is the most common mode of public transport in the region.

## Introduction

Road Traffic Incidents (RTIs) and the resultant morbidity and mortality are major public health concerns.^1,2^ The United Nations (UN) report on global road safety suggests that the global burden of RTIs is presently 1.35 million deaths while related injuries and disabilities are 50 million.^3,4^ It is reported that RTIs are the ninth leading cause of unintended mortality and morbidity worldwide.^5^ From a global perspective, the burden of road-related injuries is calculated in terms of Disability Adjusted Life Years (DALY)^6^ and is anticipated to be the fourth leading cause of morbidity by 2030.^7^ Evidence suggests that low-income to middle-income countries (LMICs’) face a disproportionate burden of RTIs, which are 1.5–3 times higher when compared to developed regions.^8,9^ It is interesting to note that LMICs account for only nearly 60% of the world’s motor vehicles but contribute to 90% of the global deaths related to RTIs.^10^ It is further postulated that sub-Saharan Africa is the leading region in terms of mortality and morbidity associated with RTIs, with the majority of related deaths and injuries occurring in pedestrians and passengers of buses, trucks, cyclists and minibus taxis.^11^

From a public health perspective, RTIs contribute significantly to the current burden of disease and mortality related to unintended injuries, which in turn contributes greatly to the current burden of non-communicable diseases in sub-Saharan Africa. In South Africa, the fatality rate of RTI’s was estimated at 25.9 per 100 000 population with a disproportionally large mortality burden associated with hospitalisations and trauma.^12^ This poses challenges in this context as health services are rendered through a re-engineered primary healthcare approach, which necessitates preventive, promotive, curative, rehabilitative and palliative healthcare to be provided to individuals, families and communities across the lifespan.^13^ The rising number of unintended road injuries, subsequent physical disability and mental health consequences attributed to RTIs places a burden on the healthcare systems in the sub-Saharan African region. This is due to medical expenditure associated with management of RTI related health complications.^5,14^ This viewpoint thus makes a unique contribution for an urgent call towards development of comprehensive, holistic and evidence-based interventions to promote health and safety in the public transport industry in light of the rising mortality and morbidity associated with RTIs in the sub-Saharan African context. Moreover, it highlights the need for implementation of multifaceted strategies underpinned by multilevel stakeholder engagement to ensure relevance for the contextual dynamics of the sub-Saharan African.

In sub-Saharan Africa, the epidemiology of RTIs remains relatively unclear, particularly in terms of precise morbidity and mortality within the diverse sub-population groups and certain geographical regions. This is because of the lack of routine health indicators and standardised data collection and reporting systems across countries in the region. The results of a systematic review of hospital-based observational studies revealed a total of 310 660 trauma patients and 99 751 RTI cases in sub-Saharan Africa as of 2015.^15^ Odonkor et al. reported that the death rate attributed to RTIs is 24.1 deaths per 100 000 populations in Africa.^3,16^ Vissoci et al. (2017) suggest that the endemic nature of RTIs in sub-Saharan Africa disproportionately affects individuals from disadvantaged socio-economic backgrounds in the region.^15^ This is further exacerbated by the limited access to healthcare and resources to adequately respond to the challenge.

There is a consensus that RTIs have consequences for quality of life from a socio-economic and public health perspective.^17,18,19,20^ The global estimated cost incurred related to RTIs is 518 billion US dollars.^21,22^ Chen et al. estimated that road injuries will cost the world economy approximately 1.8 trillion US dollars in 2015–2030.^23^ This is because of the direct healthcare costs incurred as a result of morbidity such as physical injury, disability and mental health challenges associated with RTIs.^5,24,25^ The resultant mortality and morbidity also impact general economic productivity because of the job loss associated with the negative consequences of the incidents.^26^

In sub-Saharan Africa, there is also compelling evidence of the socio-economic impact of RTIs.^15,27,28^ A study to explore the cost and disability consequences of RTI’s in Nigeria revealed that RTIs resulted in functional ramifications, which led to unemployment and socio-economic consequences.^29^ The findings of this study also revealed that RTIs resulted in disability among 29.1% of subjects in a sample of 3082 participants. Moreover, the average direct costs of healthcare were estimated to be 435 000 US dollars per annum.^29^ The finding of an earlier systematic review conducted to develop an epidemiological profile of the burden of RTIs among children and adolescents within urban areas of South Africa revealed that the mean annual incidence of urban RTI affecting children was 109, 8 per 100 000. The findings of this review subsequently highlighted the need for developing a research agenda to facilitate accurate estimates in this regard and also institute health interventions to address the challenge.^30^ Empirical evidence from other countries such as Cameroon, Nigeria and Ethiopia also reveals generally high rates of RTIs with an exponential growth in injuries and deaths being noticed with the progression of time.^28,31,32,33^ Research conducted in South Africa also alludes to the public health implications of road accidents with regard to disease burden and the number of related mortality cases. Verster and Fourie (2018), reveal that between 2010 and 2015, there were 10 613 fatal crashes and 12 944 fatalities related to RTIs in South Africa.^34^ The subsequent impact of these accidents was estimated at R 142 951 million (approximately 5 million US dollars) by the end of 2015. The findings of another study to investigate the psychosocial correlates of the impact of RTIs among South African drivers and passengers alluded to the far-reaching consequences of RTIs.^35^ Based on the findings of the aforementioned study, RTIs also have negative health outcomes that extend beyond physical injury to include mental health dimensions. These results highlight the need for comprehensive and intervention strategies to promote safety in the transport industry. These interventions must encompass promotive, preventive, curative and rehabilitative aspects.

In South Africa, the minibus taxi industry forms the backbone of the public transport industry accounting for bulk of the public mode of transportation.^36^ Statistical data reveal that the industry currently comprises 150 000 public minibus and accounts for 65% of the public transport industry.^37^ An earlier study by the Automobile Association of South Africa recorded an annual total of 70 000 minibus taxi crashes, which indicates that taxis in South Africa account for double the rate of crashes than all other passenger vehicles.^38^ Recent reports by the road traffic inspectorate on the summary of fatal crashes involving minibus taxis in Gauteng reported that between 2013 and 2016, there were 648 fatal crashes and 857 related fatalities.^39^ These fatalities included pedestrians, drivers, passengers and cyclists. The aforementioned are thus indicative of the magnitude of the problem from a public health perspective especially because resultant morbidity has an impact on quality of life.

A multitude of factors contribute to the occurrence of RTIs both from a broad public transport industry perspective and among the minibus taxis. Moreover, the consequences of these RTIs in relation to the occurrence of morbidity and mortality or a combination of both are dependent on key factors, which include physical condition of the vehicle, the extent of physical injuries and trauma, availability of emergency health care at the site of the accident and related healthcare. Van Schoor, Van Niekerk and Grobbelaar (2001) reported that mechanical failures were one of the leading causes of motor vehicle accidents in South Africa.^40^ Similar findings are also cited in studies related to the determinants of crashes and fatalities in the minibus taxi industry. Additionally, reasonable empirical evidence exists on the range of diverse factors that may be attributed to the occurrence of accidents and related complications, which are related to elements of health and safety.^41,42,43^ Contextual and international literature on the factors contributing to RTIs may be used to explain causal factors and resulting consequences of health and safety challenges within the minibus taxi industry. Evidence suggests that these relate to intrinsic factors and environmental influences. The intrinsic determinants relate to holistic health and behaviours of drivers while on the road and the external factor relate to working conditions in the minibus taxis (working hours), safety condition of taxi, and conditions of the as a driving destination.^34,38^

Taking cognisance of the global magnitude of RTIs particularly in low-income to middle-income regions, a call to action to address the challenge had been suggested so as to mitigate the public health consequences thereof.^44^ Literature suggests that effective prevention of RTI injuries including a combination of multifaceted injury prevention strategies could be effective in sub-Saharan Africa. These have typically included enforcement of relevant regulations related to speed control and compliance with other safety measures and provision of education on injury prevention interventions.^45^ In response to rising mortality associated with RTIs in minibus taxis, the department of transport in South Africa has developed several policy interventions that are oriented towards preventing accidents and safeguarding well-being of pedestrians and commuters who are often affected by these accidents. Despite legislation and initiatives directed at preventing accidents, minibus taxi-related RTIs persist to the detriment of livelihoods and the economy of South Africa and the sub-Saharan African region. As a result of the current magnitude of the problem, a deeper understanding of the health and safety challenges is required. Furthermore, intervention design should be informed by more holistic and comprehensive enquiry processes that will generate newer and inscrutable insights on the multiple realities that appear to be embedded within the notion of health and safety and the occurrence of RTIs in the minibus taxi industry in South Africa. While noticeable improvements have been made by previous interventions instituted in relation to the prevention of accidents and injuries, the lack of routine standardised data collection and evaluation approaches impedes the implementation of an effective response strategy in this regard. Historically, policies and interventions designed to address challenges of health and safety in the minibus taxi industry have excluded key influential stakeholders such as passengers, driver assistants and key informants at community level.

### Recommendations

Specific inquiry must be made to unravel and understand issues of vehicle safety, the impact of climate change and non-communicable diseases and the general work environment, behaviours and attitudes of drivers as determinants of the presently prevailing public health issues within the minibus taxi and public transport industry. This information should inform the design and implementation of multifaceted strategies that will deviate from usual programmatic approaches. Innovative approaches to routine data collection, analysis and translation should thus be adopted and intervention design should be underpinned by human centred approaches to ensure contextual relevance and effectiveness. Optimal surveillance systems also remain crucial to the provision of good epidemiological data on the nature and extent of the issue. This is essential for risk identification and analysis of emerging trends for prioritising relevant public health initiatives.^46^

Research on successful RTI prevention interventions suggests that these strategies have predominantly centred on promoting vehicle safety, improving driver behaviours and ensuring safer driving conditions.^47,48^ In this regard, successful safety interventions have included setting of driver speed limits, promoting use of vehicle safety devices and physical restraints, enforcing blood alcohol concentration levels and providing alternative forms of mass transportation.^5,9^ Moreover, policy reforms related to road safety and public transport advocate for prevention and mitigation strategies that optimise post-crash responses by improving access to emergency care and effectiveness of first line responses.^49^ Data on global best practices also suggests that countries such as Sweden, the Netherlands and Ireland have improved road infrastructure, fostered technological innovation and ensured provision of alternative forms of public transport to address health and safety challenges associated with RTIs. In the sub-Saharan African context, the adoption of technological innovation and improvement of road infrastructure for the promotion of health and safety in the public transport industry remains impeded by resource constraints related to the poor socio-economic conditions of the region. Moreover, the historical reliance on traditional forms of public transport mainly in the form of minibus taxis may pose challenges for introduction of newer and alternative forms of transportation in terms of public acceptability.

Nonetheless, the escalating levels of mortality and morbidity associated with RTIs in the public transport industry, particularly with the influence of minibus taxis require urgent intervention that is informed by comprehensive, integrated and evidence-based approaches. In this regard, a contextual understanding of the multiplicity of contributing factors is required through the use of innovative data systems and multilevel stakeholder engagement, co-design, implementation and collaborative monitoring to ensure success of prevention and mitigation strategies. The application of human-centred research designs and collaborative engagement of scholars, policymakers, various sectors of government, community members, industry leaders and public transport service providers may be instrumental for ensuring holistic and contextually relevant interventions that are designed for implementation.
